# The need for cybersecurity self-evaluation in healthcare

**DOI:** 10.1186/s12911-024-02551-x

**Published:** 2024-05-23

**Authors:** Wendy Burke, Andrew Stranieri, Taiwo Oseni, Iqbal Gondal

**Affiliations:** 1 Global Professional School, Federation University, PO Box 663, Ballarat, 3353 Victoria Australia; 2 Institute of Innovation, Science and Sustainability, Federation University, PO Box 663, Ballarat, 3353 Victoria Australia; 3 School of Computing Technologies, RMIT University, GPO Box 2476, Melbourne, 3001 Victoria Australia

**Keywords:** Cybersecurity, Healthcare, Self-evaluation, Empowerment

## Abstract

The Australian healthcare sector is a complex mix of government departments, associations, providers, professionals, and consumers. Cybersecurity attacks, which have recently increased, challenge the sector in many ways; however, the best approaches for the sector to manage the threat are unclear. This study will report on a semi-structured focus group conducted with five representatives from the Australian healthcare and computer security sectors. An analysis of this focus group transcript yielded four themes: 1) the challenge of securing the Australian healthcare landscape; 2) the financial challenges of cybersecurity in healthcare; 3) balancing privacy and transparency; 4) education and regulation. The results indicate the need for sector-specific tools to empower the healthcare sector to mitigate cybersecurity threats, most notably using a self-evaluation tool so stakeholders can proactively prepare for incidents. Despite the vast amount of research into cybersecurity, little has been conducted on proactive cybersecurity approaches where security weaknesses are identified weaknesses before they occur.

## Introduction

Healthcare is considered one of the most targeted sectors for cybersecurity breaches [1, 2]. Since the Australian Notifiable Data Breach scheme began, the healthcare industry has consistently reported more data breaches overall than other industries [3]. As shown in Table 1, between January 2019 and December 2023, 942 health sector notifications were received under the ‘Notifiable Data Breaches’ scheme [4]. Further, this excludes breaches under the My Health Records Act 2012. According to the Office of the Australian Information Commissioner, there were an additional 42 data breaches to the My Health Record system in 2017/2018 [5].

Of the 164 health service incidents reported between Jul - Dec 2022, 87 incidents resulted from malicious or criminal attacks, and the rest were caused by human error or system faults [6, 7].

**Table 1:** Notifiable healthcare data breaches in Australia

Notification period	Overall number of notifications	Health information notifications	%
Jan - Jun 2019	459	105	23
Jul - Dec 2019	537	117	22
Jan - Jun 2020	518	115	22
Jul - Dec 2020	539	123	23
Jan - Jun 2021	446	85	19
Jul - Dec 2021	464	83	18
Jan - Jun 2022	396	79	20
Jul - Dec 2022	497	71	14
Jan - Jun 2023	409	60	15
Jul - Dec 2023	483	104	22
**Total**	**4748**	**942**	**20**

In addition, the health sector remains the top reporter of data breaches. In the July to December 2023 reporting period, the health sector reported 104 breaches, with the next closest sector being finance, with 49 breaches [7]. A 2020 Health Sector Snapshot [8] speculated that COVID-19 changed the threat landscape of Australia’s healthcare sector, making it a more significant target. Changes to working environments, including the shift to working at home and increased use of telehealth, increased and exposed the sector to new vulnerabilities. The Australian Cyber Security Centre [9] released a critical alert due to advanced persistent threats to the healthcare sector. However, under-reporting, even when disclosure is legally mandated, is prevalent, according to the Australian Cyber Security Centre [10].

Australia’s National Digital Health Strategy outlines that Australian healthcare consumers and providers want, and are ready for, digital access to health services as long as their health information can remain confidential and secure [11]. Much research has already been undertaken in designing healthcare security models, most recently in electronic health records and using blockchain [12–14]. However, little research has been conducted into the healthcare sector’s capacity to assess its cybersecurity capability to take a proactive approach to cyber threats [15].

Proactive cybersecurity refers to measures taken in advance to prevent or discourage an attack. This includes implementing security protocols, monitoring systems, and educating stakeholders to identify and mitigate potential vulnerabilities. Reactive cybersecurity, conversely, refers to measures taken in response to an attack that has already occurred [16, 17].

Implementing proactive cybersecurity measures helps individuals, organisations and sectors (such as healthcare) gain a sense of empowerment. With a strong focus on individuals gaining mastery of their lives or situations (e.g. being allowed to be part of the solution), empowerment gives the necessary capacity to increase individual expectancy of success [18]. First theorised by Rapparort [18], empowerment strongly focuses on individuals gaining mastery of a situation and gives the necessary capacity to increase their expectancy of success. In cybersecurity, individuals need to be informed about threats and equipped with the knowledge and tools to mitigate attacks. While cybersecurity specialists can implement and oversee an organisation’s security system, participation by individuals is crucial to ensure these processes work effectively [19].

However, human error and lack of awareness can still bypass even the most sophisticated technical safeguards. A cyberattack on a healthcare organisation can have immediate and severe consequences. A review of a 2020 cyberattack impacting the University of Vermont Health Network found that within minutes, the healthcare organisation lost all network intranet services, clinical systems (including laboratory, pathology, pharmacy and radiology systems), email communications and their electronic medical record system [20]. Systems did not return online for almost one month, yet the healthcare organisation still needed to provide patient services. The disruption was found to have been caused by an employee opening a personal email infected with malware on a health organisation laptop.

In Australia, healthcare insurer Medibank Private revealed in 2022 that it had detected “unusual activity” on its network. On the 1^st^ of December, Medibank Private publicly announced that files containing sensitive personal and healthcare claim data from 9.7 million current and former customers were released on the dark web [21]. News articles reported that stolen credentials from a high-level Medibank staff member resulted in the breach [22]. Both of these attacks were not overly sophisticated or technical. In the case of the cyberattack at the University of Vermont Health Network, it was reported that the email, whilst from a legitimate local business that had been hacked, was not specifically targeted towards the health service [23]. Medibank Private has yet to confirm how the credentials were stolen. Still, it appears criminals could access confidential information with little effort by accessing legitimate login details.

This underscores the need for a strong cybersecurity culture that goes beyond technical solutions. Understanding how cultural, structural and technological factors affect the health sector and impact cybersecurity initiatives is critical for designing, implementing and disseminating protective strategies [24].

However, empowering individuals within the healthcare sector requires more than just awareness training. Generic cybersecurity solutions, designed for a broad range of industries, often overlook the unique vulnerabilities and complexities faced by healthcare organisations. Unlike other sectors, healthcare organisations are extraordinarily complex to secure against cyber threats [15, 25].

According to Thompson [26], the increased use of information technology and the associated collection and use of data is placing new pressure on the healthcare sector not previously seen. Cybersecurity has not previously been considered a primary role within the industry. Healthcare computer security personnel have to deal with old, legacy systems, lack of funding, lack of cybersecurity personnel and health staff using workarounds to intentionally or unintentionally boycott security [27].

Despite these significant challenges, empowering those within the healthcare sector has the potential to overcome these obstacles. When individuals feel empowered, they are equipped with the knowledge and tools to recognise cyber threats [28, 29]. This includes the ability to identify suspicious emails, recognise phishing attempts, and understand best practices for data handling. This sense of ownership and responsibility fosters a collective defence against cyber threats, complementing technical security measures [30].

However, to effectively empower the healthcare sector, we need a deeper understanding of its specific cybersecurity challenges and needs. Few studies in the Australian context have examined the interplay of diverse factors that impact cybersecurity in healthcare.

The aim of this research is to investigate the applicability of general trends in healthcare cybersecurity to the specific context of Australia. We will achieve this by examining recent literature reviews that identify vulnerabilities, challenges, and potential solutions within the healthcare cybersecurity landscape globally. Following this, a focus group will be conducted to explore these established trends in the context of the Australian healthcare system. This two-pronged approach will allow us to assess how well-documented cybersecurity threats translate to the Australian environment, ultimately informing the development of practical strategies and tools specifically tailored to empower the Australian healthcare sector.

## Literature survey

The healthcare sector has undergone a significant digital transformation in recent years. This is partly due to the rise of the internet and digital healthcare tools and technologies [31]. These digital advancements and growing reliance on interconnected healthcare systems have also created a new set of vulnerabilities. Sensitive patient data, including medical records, financial information, and personal details, has become a prime target for cybercriminals.

This surge in cyberattacks on the healthcare sector poses a significant threat to patient privacy, financial stability, and even the quality of care [32, 33]. Data breaches can disrupt critical services, delay diagnoses, and compromise patient safety. The financial consequences for healthcare providers can be severe, including hefty fines, reputational damage, and the cost of remediation.

Several researchers have examined literature on the cybersecurity landscape within healthcare that shed light on vulnerabilities, challenges, and potential solutions. To gain the most up-to-date understanding of this evolving field, we have focused specifically on literature reviews published within the past three years. The criteria for research article selection is described in Table 2.

**Table 2:** Research criteria

**Data Source**	Google Scholar
**Time Frame**	2020 to Now (April 2024)
**Document Type**	Journal articles
**Keywords**	“healthcare” OR “health care” OR “health” AND “cybersecurity” OR “cyber” AND “literature review” OR “review”

Google Scholar was used to identify relevant literature reviews. Focusing on recent reviews ensured that the analysis incorporated the latest findings and addressed the most pressing cybersecurity challenges facing healthcare institutions today. By synthesising these reviews, this work will assist in understanding current vulnerabilities and research gaps in healthcare cybersecurity. Table 3 describes a summary of the selected research articles.

**Table 3:** Overview of papers reviewed

Author(s)	Year	Title	Papers Reviewed	Date Range	Search Criteria
Vilakazi and Adebesin [34]	2023	A systematic literature review on cybersecurity threats to healthcare data and mitigation strategies	41	2017 to March 2022	(“cyber”) AND “threat*” OR “attack*” OR “crime*”) AND (“health*”) AND (“data”) AND (“secur*” OR “mitigate” OR “protect”)
Aldossri and Rahman [35]	2023	A systematic literature review on cybersecurity issues in healthcare	21	2019 to 2022	(security or cybersecurity) AND healthcare AND (threats OR vulnerabilities OR challenges)
Herrera et al. [36]	2023	Cybersecurity in health sector: a systematic review of the literature	71	2018 to 2022	“cybersecurity” “cyberattacks” “health” and “hospital”
Nifakos et al. [25]	2021	Influence of human factors on cybersecurity within healthcare organisations: A systematic review	70	2010 to 2021	Human AND (Cybersecurity AND “training” OR “Information security awareness” )
He et al. [37]	2021	Health care cybersecurity challenges and solutions under the climate of COVID-19: Scoping review	56	2011 to 2020	(covid OR healthcare) AND cybersecurity.
Sardi et al. [38]	2020	Cyber-risk in health facilities: A systematic literature review	84	1992 to 2020	“Cyber” OR “Computer security” AND “Health” AND “Risk”

Vilakazi and Adebesin [34] conducted a systematic literature review to explore mitigation strategies for cybersecurity threats targeting healthcare data. The review identified several critical vulnerabilities in healthcare cybersecurity. These included the rise of new digital technologies, a lack of established cybersecurity policies, human factors like staff unawareness, and limited consensus among stakeholders regarding cybersecurity challenges. Additionally, the review found inadequate investment in cybersecurity measures.

While Aldossri and Rahman [35] identified similar cybersecurity challenges in healthcare as Vilakazi and Adebesin [34], their research identified several key areas of concern in healthcare cybersecurity: human factors, technological vulnerabilities, data security and privacy, insider threats, and external attacks. The studies reviewed highlighted the critical need for staff training and awareness programs.

Aldossri and Rahman [35] concluded that overall, there had been a significant increase in cyberattacks targeting healthcare institutions, and there was no sign of this trend subsiding. This trend underscores the critical need for proactive measures, with the researchers suggesting that the most effective approach to developing and sustaining strong cybersecurity within healthcare lies in the persistent education of staff and patients.

Sardi et al. [38] literature review strengthens the themes raised by the previous two literature reviews [34, 35] regarding the growing cyber-risk in healthcare. Their analysis identified four key themes: human actions, system failures, internal process breakdowns, and external events. This aligns with the highlighted vulnerabilities, emphasising the need for proactive solutions.

Sardi et al. [38] also highlight two critical gaps in the research: a lack of comprehensive studies on cyber-risk assessment in healthcare and the lack of research in countries other than the United States. These gaps leave the healthcare sector vulnerable. Without comprehensive cyber-risk assessments, healthcare organisations lack a complete picture of their vulnerabilities (or strengths). This makes prioritising mitigation strategies challenging and exposes them to potential attacks.

Similarly, focusing research primarily on the US healthcare system overlooks the unique challenges healthcare sectors face in other countries. Healthcare systems differ significantly around the globe, with infrastructure, staffing, and technological adoption variations. These disparities can substantially impact the types and severity of cyberattacks faced by healthcare institutions.

While Hu et al. [37] literature review covered articles from 2011-2020, the main focus was on examining cybersecurity challenges in healthcare during COVID-19. The COVID-19 pandemic exposed a glaring vulnerability in healthcare cybersecurity: the unpreparedness for a large-scale shift to remote work. These findings align with the observations of Herrera et al. [36]. Healthcare organisations, forced to adapt quickly, lacked the planning and the cybersecurity measures necessary to secure this new environment. This resulted in a myriad of critical challenges, such as insufficient business continuity plans, which meant disruptions could significantly impact essential services. The lack of security awareness training among healthcare staff increased their vulnerability to cyber threats in this unfamiliar remote work environment. These weaknesses and limited experience in remote work among staff made the sector a prime target for cyberattacks like malware.

Building upon the vulnerabilities exposed by the COVID-19 pandemic [36, 37], which focused on the need for long-term planning and leveraging best practices from other sectors, a systematic review conducted by Nifakos et al. [25] aimed to identify recurring human behaviours that weaken the cybersecurity position of healthcare organisations. Human factors emerge as a recurring theme in the healthcare cybersecurity literature reviewed for this paper [34–36, 38].

Consistent with observations from previous reviews already mentioned, Nifakos et al. [25] highlighted the critical role of training and awareness campaigns in combating cyberattacks and the need for regular assessments to identify and address security gaps within healthcare systems. For example, healthcare IT personnel should be equipped to detect social engineering attempts, while healthcare professionals should learn to recognise them.

Additionally, despite the growing recognition of the need for cybersecurity training, healthcare organisations often lack dedicated security leadership. The absence of roles like Chief Information Security Officer, as highlighted by Nifakos et al. [25], weakens defences. This creates a crucial gap. A coordinated effort is necessary to promote good cybersecurity practices.

The healthcare sector’s digital transformation has brought immense benefits but also introduced new cybersecurity challenges. Increased reliance on interconnected systems exposes sensitive patient data to cyberattacks, potentially jeopardising patient privacy, financial stability, and even the quality of care.

Vulnerabilities, including technological advancements, human factors like staff unawareness, and inadequate investment in cybersecurity measures, have been observed in recent literature [25, 34, 35, 38]. The COVID-19 pandemic further highlighted the sector’s unpreparedness for a large-scale shift to remote work, exposing weaknesses in planning and security awareness [36, 37]. However, existing research often focuses on global trends, with limited exploration of how these challenges manifest and require solutions in specific healthcare system locations. Given Australia’s unique healthcare landscape, a focused examination of the current state of cybersecurity in this sector is warranted.

Furthermore, research gaps necessitate further studies on comprehensive cyber-risk assessment methodologies and best practices applicable to diverse healthcare systems worldwide [38]. One potential approach could involve the creation of a self-assessment tool that caters to the specific needs of different healthcare actors. This tool could be designed with multiple sections, allowing those within the healthcare sector to assess their cybersecurity position effectively.

This research aims to bridge this gap by assessing the applicability of the general findings from current literature to the Australian healthcare sector. Australia’s unique healthcare landscape, characterised by a mix of public and private providers, an emphasis on digital health initiatives, a national framework for health information privacy and security, and an Australian-wide health record system, may necessitate tailored cybersecurity strategies. By examining the general vulnerabilities identified in the literature and facilitating a focus group discussion to evaluate their relevance to the Australian context, this research will provide valuable insights to strengthen cybersecurity preparedness within the Australian healthcare sector.

## Method

This study employed a semi-structured focus group discussion to gain in-depth insights into the current state of cybersecurity in the Australian healthcare sector. While large-scale surveys can provide a broader overview, focus groups offer a distinct advantage for exploring complex issues. As Crone [39] highlights, semi-structured focus groups utilise open-ended questions that not only elicit information but also allow participants to draw on personal experiences that may not have been previously considered in research. This approach is particularly valuable in the Australian healthcare context, where the interplay between cultural, structural, and technological factors influencing cybersecurity remains under-investigated.

By convening a small group of key stakeholders that represented government, healthcare providers, and cybersecurity experts, we fostered a rich discussion into the unique challenges and opportunities specific to the Australian healthcare cybersecurity landscape. The focused nature of the group, as Krueger and Casey [40] point out, allowed for a more nuanced understanding of participants’ perspectives and experiences. Interaction between participants, a hallmark of focus groups, fosters the generation of insights that would be less accessible with individual interviews [40, 41]. Furthermore, focus groups facilitate discussions from diverse viewpoints, helping us understand the “why”, “how” and “what” of healthcare cybersecurity without pressuring participants towards a consensus [42, 43].

While the information gathered from focus groups is primarily representative of the participating group and cannot be statistically generalised to the wider healthcare sector, it offers a valuable platform for participants to compare and contrast their experiences and views regarding the sensitive topic of healthcare cybersecurity. This exchange of knowledge and perspectives, along with insights gleaned from the existing literature, allowed for a richer understanding of the current state and future directions for fortifying cyber-defences within the Australian healthcare sector.

### Participants

To ensure diverse perspectives, participants were recruited based on their involvement in one or more of the following areas: the Australian healthcare industry, cybersecurity practice, or cybersecurity research. Twelve organisations with ties to the Australian healthcare sector were sent email invitations. Five participants responded and took part in the focus group discussion led by two of the authors.

The participants had varied backgrounds; however, most had a specialised interest in computer security and healthcare. This purposeful selection strategy ensured participants understood the cybersecurity complexities within the Australian healthcare sector.

The size of the focus group was a deliberate choice. Because the topic of healthcare cybersecurity can be sensitive, fostering a safe space for open and honest discussion was paramount. A smaller group setting allows participants to feel more comfortable sharing personal experiences and insights, leading to a richer and more nuanced understanding of the challenges and opportunities in this critical area.

All participants in this focus group possessed a deep understanding of the Australian healthcare environment and its security landscape. To protect participants’ identities, each was given a pseudonym. Participant 1, a Chief Information Officer for a public regional healthcare service in Australia, brought firsthand experience managing cybersecurity within the healthcare system. Participant 2, a general practitioner (GP) in Victoria and a member of the Royal Australasian College of General Practitioners (RACGP) Expert Committee - Practice Technology and Management, offered a practitioner’s perspective on technology and security challenges. Participants 3 and 4 brought a unique perspective as senior university researchers with extensive experience in the healthcare sector, particularly in leading healthcare-specific projects. Participant 5, with experience at an industry-university cybersecurity research centre, provided expertise in security best practices.

### Procedure

The focus group was conducted simultaneously face-to-face and via teleconferencing. Both delivery methods were used to make it easier for the participants to take part regardless of their physical location.

The objective of using a focus group was to elicit in-depth insights into issues identified by participants. Two researchers facilitated the focus group. One researcher acted as the convener, and the second took notes. Researchers and two participants were seated around a table, while the other three were connected via Skype.

Table 4 outlines the structure of the focus group. The focus group used a four-stage structure to explore participant perspectives of cybersecurity and its impact on the Australian healthcare sector. The first stage was an introduction, welcoming participants and outlining the session’s purpose. This initial phase aimed to establish rapport and ensure participants understood the discussion’s direction.

**Table 4:** Structure of the focus group

Stage	Aims	Approximate Duration
1: Introductions	Welcome participants, introduce facilitator and purpose of the session	10 mins
2: Impact	Understand the perspectives of participants on how cybersecurity threats and solutions affect the Australian healthcare sector	30 mins
3: Guided impact	Explore how participants perceive the influence of specific cybersecurity vulnerabilities and opportunities on the Australian healthcare sector	40 mins
4: Wrap-up	Summarise key points, ask final questions, thank participants	10 mins

The second stage delved into participants’ general understanding of the impact of cybersecurity on the Australian healthcare sector. Open-ended questions encouraged participants to discuss how cybersecurity affects healthcare services, professionals, and consumers (see Table 5). This stage aimed to understand their concerns and experiences with cybersecurity in the healthcare context.

The third stage transitioned to a more guided discussion. Drawing on themes identified from the literature review on cybersecurity and healthcare, the facilitator presented specific topics for participant commentary if they had not organically arisen in the second stage. These topics included the impact of legacy systems, increased medical device technology, organisational and societal culture, media reporting of threats, IT security budget and support, and the black market value of healthcare data (see Table 5). This stage aimed to gain deeper insights into how participants perceive these themes influencing cybersecurity vulnerabilities and opportunities within the Australian healthcare system.

The last stage served as a wrap-up. Key points emerging from the discussion were summarised, allowing participants to confirm their understanding and providing an opportunity for final thoughts. Participants were also introduced to the concept of a healthcare cybersecurity index-a self-assessment tool for the Australian healthcare sector-to gauge their initial thoughts. This stage aimed to solidify the key findings obtained throughout the focus group.

It is important to note that indicative questions used in Stages 1 and 2 to guide the initial open-ended discussions were also derived from the healthcare cybersecurity literature (see Table 5). This ensured a thematic consistency throughout the focus group and allowed for a comprehensive exploration of participants’ understanding and experiences with cybersecurity in the Australian healthcare context.

**Table 5:** Indicative questions used in the focus group

Stage	Indicative Questions
2: Impact	What impact does cybersecurity have on the healthcare sector in Australia from the point of view of healthcare services, professionals and consumers?
3: Guided Impact	What impact do you think the following has on cybersecurity and healthcare in Australia?
	The role of legacy systems/aging technology?
	The changing landscape of medical technology, e.g. increase the use of wireless medical devices?
	Organisational culture/societal culture?
	The role the media play when reporting attacks and threats regarding healthcare and cybersecurity incidents
	Budgets and support for IT security professionals in healthcare organisations?
	Education/training/professional development?
	The ‘black market’ value of healthcare data?

The entire focus group session was audio-recorded with participant consent to ensure comprehensive data capture for analysis. The recordings were transcribed verbatim, preserving and allowing for a nuanced examination of participant perspectives and a richer understanding of the group’s overall discussion.

The primary function of the focus group was to allow participants to discuss the general vulnerabilities identified in existing research and literature with the aim of providing a foundation. Secondly, the focus group facilitated a crucial discussion on how these established vulnerabilities translate to the specific context of Australian healthcare.

### Ethical considerations

Ethical approval was obtained from the Human Research Ethics Committee of Federation University (Project Number B19-087), and all participants gave written informed consent prior to participation.

### Establishing themes

The lead researcher audio-recorded and transcribed the focus group verbatim. They also checked the transcripts for accuracy against the audio files, correcting and adding any inaccuracies or missing words.

The focus group transcript and interviewer notes were organised and coded using QSR NVivo 20 and analysed using thematic analysis. Thematic analysis has become a prominent method in recent qualitative cybersecurity research (e.g. [44, 45] and was used as described by Braun and Clarke [46]. Braun and Clarke’s [46] reflexive thematic analysis approach acknowledges the researcher’s subjectivity as a strength and leverages it to uncover deeper meanings within the data. Another advantage is the researcher’s insider perspective (having experience with the Australian healthcare sector and cybersecurity), which facilitated dialogue and trust among participants. The six steps of the thematic analysis process are detailed in Table 6.

**Table 6:** Applying reflexive thematic analysis to the focus group transcript

Stage	Process
1: Data familiarisation	Immersion into the transcript and audio recording, taking notes.
2: Code generation	Begin assigning codes to segments of the text in the transcript.
3: Theme construction	Start grouping codes into broader themes.
4: Theme review	Critically assess the themes developed by going back to the transcript. Consider your own understanding and its influence, and relationships between themes and the wider context of the research.
5: Theme definition and naming	Refine themes by giving them clear and concise names that accurately reflect their content.
6: Producing the report	Write a clear and concise report that describes the thematic analysis process.

The lead researcher completed the initial coding. Next, two researchers reviewed the coding, and a discussion was held between all three researchers. This collaborative approach aimed to refine the codes, ensure applicability and develop a shared understanding of the data. The codes were also re-examined for overlap, and those closely related were consolidated. Following the review and refinement of the initial codes, the frequency of each code was calculated. This analysis helped identify the most prominent themes within the data. These initial themes were constructed by the lead researcher and then reviewed and discussed with the other researchers. This review included the lead researcher critically assessing the developed themes in light of the entire transcript. Notably, some focus group responses overlapped with multiple themes. Finally, the research team collectively refined the themes to ensure they accurately captured the nuances of the data. The final themes and sub-themes can be found in Table 7.

## Findings

The discussions revealed a multifaceted landscape of challenges and opportunities. Key themes emerged around the unique vulnerabilities of the healthcare environment, the financial challenges of cybersecurity, the need to balance privacy and transparency and the role of education and regulation. Participants also highlighted the critical shortage of IT security specialists, the limitations of legacy systems, and the ever-evolving threat posed by sophisticated cyberattackers. The role of the media in raising awareness of cyber threats was discussed, with both positive and negative aspects considered. Finally, participants emphasised the need for a proactive approach to cybersecurity, stressing the importance of preparedness and ongoing vigilance.

Table 7 provides a high-level overview of the summarised comments. Each theme is presented in more detail below, with quotations to demonstrate findings.

**Table 7:** Summary of the research findings

Theme/sub-themes	Summarised responses
**The Australian healthcare landscape**	
Complexities	Physical environment
	Complex workflows
	Complexities of data access involving numerous actors (patients, providers, government agencies)
	Legacy systems and software continue to be used
	Differing priorities between private and public healthcare organisations
	Medical equipment is increasingly connected to networks
Balancing security & usability	Growing awareness of cyber threats among healthcare professionals
	Clinician time constraints impacting security practices (logging in/out)
	Convenience-oriented practices (e.g. non-expiring passwords)
Regulatory & educational gaps	Public confusion and lack of understanding about data security
	Lagging legislation that struggles to keep pace with evolving technologies
**The financial challenges of cybersecurity in healthcare**	
Funding & resource allocation	Difficulty for healthcare providers to raise fees to cover cybersecurity expenses
	Balancing the need for updates with budgetary limitations
	Public funding models limiting resources for ongoing maintenance
	IT security seen as an indirect cost, competing with direct clinical service needs
	Perception that cybersecurity measures are expensive and may not be fully effective
	High cost of hiring IT security specialists
Burden of legacy systems	High costs associated with replacing outdated systems due to interoperability challenges
	Healthcare legacy systems are a major vulnerability point
**Balancing privacy and transparency**	
Balancing privacy & data security	Importance of maintaining patient privacy and confidentiality
	Potential for patient information to be misused
	High value of patient data on the black market
Public discourse	Challenges with media communication during healthcare cyberattacks
	Importance of media coverage in raising awareness and promoting best practices
	Transparency as a key factor in minimising harm and encouraging debate
	Public discourse as a driver for change on social issues
Patient trust & data sharing	Lack of public understanding of healthcare data storage and access
	Public fear of cyberattacks leading to opting out of electronic health records
**Education and regulation**	
User education & awareness	Importance of effective user training/education
	Media attention to cybercrime can stimulate user education
	Users often lack understanding of cybersecurity risks
	Users sometimes prioritise convenience over security (e.g. weak passwords)
	Importance of building a resilient system to minimise the impact of attacks
Role of regulation and enforcement	Need for clear legislation defining roles, responsibilities, and consequences for cybersecurity breaches
	Importance of a monitoring authority to ensure enforcement of cybersecurity standards

### The Australian healthcare landscape

The cybersecurity landscape in healthcare faces unique complexities. Legacy systems remain operational due to the critical role they play in patient care. Upgrading these systems can be a costly endeavour, and the budgetary constraints faced by public health organisations often leave them lagging behind the private healthcare sector, which have more resources to invest in robust security measures. By consensus, the focus group suggested that there were, and still are, legacy systems within the Australian healthcare sector. However, the reasons for using legacy systems are more than just the cost of upgrading. Participant 2 explained that “*Windows XP was held on many peripheral computers throughout GP offices...way longer than was being supported by Windows*” because they were comfortable with the system. Participant 3 concluded that “*the natural inclination of most people, who are not IT geeks, is to use an old system and keep using it*”.

Additionally, the increasing integration of medical devices into networks creates new attack points. With the introduction of the Internet of Things (IoT) and BYOD (Bring Your Own Device), healthcare services inadvertently open themselves up to potential cyber-incidents. “*BYOD was always considered a good thing to help with hospitals’ bottom line because everyone can bring in their own hardware*” (Participant 1). The trade-off is that the healthcare service cannot ensure that the BYOD device has antivirus software and appropriate cybersecurity controls.

The physical environment itself poses challenges. “*It is not like the banking sector....we have an open door policy welcoming people. Anyone can walk into any public health service and plug something in*” (Participant 1). The open nature of health services, especially hospitals, makes it difficult to secure entry points and control device usage. Participant 3 stated, “*nurses often talk about the security of health information in hospitals or the lack of it because hospitals were actually not designed in a way that encourages security*”. “*...you do not necessarily have security zones or necessarily physical access controls to terminals in hospitals*” (Participant 5). This highlights a fundamental conflict between the open and welcoming nature of healthcare and the need for robust cybersecurity measures. It also creates a significant challenge for healthcare providers who need to safeguard sensitive patient data while maintaining an accessible environment for patients and visitors.

Additionally, the complex healthcare workflow can lead to delays in detecting and responding to cyberattacks, increasing the potential for damage. When discussing three recent large attacks on regional Victorian healthcare providers, Participant 1 acknowledged, “*it could be some months before we actually really understand what happened and the extent of the damage they did*”. Participants also acknowledged that the public and healthcare professionals seem to be aware of these vulnerabilities, potentially leading to a loss of trust in the healthcare system’s ability to safeguard personal information.

There are a vast number of stakeholders accessing healthcare data, from patients to healthcare providers and government agencies; all of this further complicates access control. This widespread access, however, is often accompanied by a lack of understanding of cybersecurity best practices among these stakeholders; especially consumers. When reflecting on the uptake of MyHealthRecord, Participant 3 remarked that “*a number of people I know opted out, and I asked them why, and they couldn’t tell me. It was just the thing to do*”.

Finally, outdated legislation and historical resistance to disruptive technologies within the healthcare sector have resulted in a legacy infrastructure that may not be well-equipped to handle modern cybersecurity threats, which was identified as a unique aspect of the Australian healthcare landscape. Participant 1 emphasised that “*legislation is lagging in all this stuff as well, we’re still using legislation from some time ago - it can’t keep up with all the technologies*”. Current security practices can also be misguided, with a focus on clinician convenience through non-expiring passwords and free guest Wi-Fi to be consumer-centric, inadvertently creating vulnerabilities. As Participant 1 pointed out, it’s “*convenience versus security....*[you stop] *worrying about how the doctors login by giving them passwords that don’t expire*”.

This convergence of outdated technology, varying financial priorities, and the growing network dependence on internet-ready medical equipment creates a challenging environment to effectively safeguard against cyberattacks.

### The financial challenges of cybersecurity in healthcare

While focus group participants acknowledged the importance of robust cybersecurity, they did reveal that it had a significant strain on healthcare organisations, particularly publicly funded organisations. For example, on the one hand, a General Practice (GP) is a private business; on the other hand, it is a community health service. A private business can offset the cost of cybersecurity protection with the amount charged for services or products. With GPs however, having maximum cybersecurity protection is challenging as Medicare (Australia’s universal healthcare system) prescribes reimbursement limits on services. For example, Participant 2 stated, *“cybersecurity becomes a very difficult and challenging business operational issue when you cannot put your fees up to compensate for this added expense”*. *[Healthcare professionals] “are aware of a lot of security issues but also report that they do not have (cannot afford) the capability of doing anything differently because they do not have the physical setups to do so*” (Participant 3).

Another factor mentioned in the discussion was that most healthcare organisations have reasonably small networks and rely on third-party IT providers to update and protect their systems. How much is paid for that service impacts the level of protection received in terms of hardware and software updates. Even extensive healthcare services are constrained due to funding streams. “*We’d love to update everything, but there is a significant dollar perspective that needs to be considered*” (Participant 1).

Building a skilled cybersecurity workforce adds another layer of financial pressure. The cost of IT specialists often exceeds existing budgets allocated for cybersecurity. Participant 1 commented that, unlike the private sector, public healthcare providers rarely make surpluses and: *“the cost (of IT specialists) is well in excess to what we have allocated for”*. For both participants currently employed in the healthcare sector, investment in IT security specialists is, at times, better spent on employee education. “*You have an organisation with 4,500 employees; it only takes one employee to click on a link or give their credentials to another person*” (Participant 1).

Finally, participants emphasised the need for increased government funding to address the financial challenges of healthcare cybersecurity. The *“....Federal Government needs to look at seriously funding it and what we going to do about it because the problem isn’t going to go away. There is no way GPs on their own, GP surgeries or small medical businesses are going to be able to cover these costs”* (Participant 2).

### Balancing privacy and transparency

The focus group discussions highlighted the tension between privacy, the need for transparency in the wake of cyberattacks and trust.

There was an extensive discussion on the importance of patient privacy and the impact cybersecurity breaches have on the individual consumer and the healthcare organisation. Maintaining patient privacy and confidentiality is legislated in Australia. Participant 2 acknowledged, “*we are holding some elements of patient information that can be useful if you want to use them for nefarious reasons.*” Participant 1 indicated that “*health is targeted unlike any other sector...because we collect a lot of personal information...that personal information is worth more on the black market than credit card information if you can sell it*”. “*...generally there is a lack of understanding from healthcare users about how their healthcare data is being stored and accessed*” (Participant 3). Participant 4 indicated that “*privacy concerns are certainly important to the patient, but it is not as immediate to the healthcare provider*.”

A level of secrecy surrounds cyberattacks in the healthcare sector. Participant 1 was not authorised to respond to the question concerning the media’s role in reporting attacks. Instead, they stated that the healthcare service has very clear policies that assert that the CEO or communications director can only speak to the media. They also mentioned that from a consumer’s perspective, they could see the need to be aware of cyberattacks: “*the more we know about it, the more it is not a huge issue. Attacks will occur, so we must be aware of it in some form*” (Participant 1).

Challenges with media communication were also raised. While some participants emphasised the importance of transparency in informing the public and encouraging system improvements, others expressed concern that media coverage could damage public trust or pressure healthcare systems in unhelpful ways. Participant 3 commented that “*people hear about attacks in the media, then they suddenly opt out of healthcare records because they believe their medical history cannot be protected*”.

The focus group also discussed the need to balance transparency with potential negative consequences. Open communication about cyberattacks can incentivise improvements and raise public awareness. However, overly sensationalised media coverage could lead patients to opt-out of data sharing, hindering healthcare delivery.

### Education and regulation

Participants acknowledged that healthcare is a prime target for cyberattacks, yet many organisations remain in a reactive mode. The sophistication of these attacks necessitates a more proactive approach.

Educating healthcare professionals and patients alike emerged as a critical strategy. Participants emphasised the importance of user awareness regarding cyber threats and best practices for protecting sensitive data. As Participant 2 stated “it’s all very good to say to the owners of the business, or the managers of the hospital or the community health centres or the patient we’re working with that they need to be cybersecure but unless they’re trained it doesn’t work - that leaves the door open for attacks”. This then raised the point that effective cybersecurity strategies must address not just technology solutions, but also users understanding and behaviour.

All participants agreed that the media’s role in making healthcare cybersecurity public discourse is indispensable. Participant 2 stated, “*if the media can be used to stimulate the clinicians and other users of the system to get more skilled at protecting the system, then that is a good thing.*” Transparently and openness about a cyberattack that has occurred is essential because it has the potential to help prepare other healthcare organisations. It can also “*put some pressure on Ministers to work on policy, increase funding and improve things from that point of view*” Participant 5.

Updating existing legislation and regulations to reflect the evolving cyber threat landscape was seen as crucial. Clear definitions of roles, responsibilities, and consequences for non-compliance are necessary. However, highlighting the complexity of the issue, Participant 5 commented “*what standards are in place at the federal, state and organisational level and is there a regulator that is responsible for monitoring compliance of those standards and are those standards fit for purpose?*”.

Participants spoke candidly about the fact that there were no simple solutions to the complex challenge of healthcare cybersecurity. “*Health is being targeted. We can see that through all of our tools that are protecting and detecting against unauthorised access, but we are very much in a reactive mode*” (Participant 1). Participant 2 stated that “*...most practices will be trying to protect themselves...how effective that is, is a good question, and how to measure the effectiveness is an excellent second question.*” Furthering this statement, “*we need to ensure that cybersecurity is beefed up in such a way that at a consumer level, and at a professional level, the trust is maintained and even enhanced*” (Participant 4).

The focus group did, however, acknowledge the financial burden of implementing robust cybersecurity measures, particularly proactive monitoring. Participant 3 suggestion that “*there is a need for a very strong, very public and mainstream sort of framework, that is actually visible enough that it explains what it is there for to the general public as well as what it is there for and what the requirements are for the medical people*” was agreed upon by the whole focus group especially if it was Australian focused. This framework would provide clear guidelines and resources for both the general public and healthcare professionals in the healthcare sector.

## Discussion

The findings highlight the significant cybersecurity challenges faced in healthcare. Outdated systems remain operational due to their critical role and user familiarity despite the security risks. Limited budgets, particularly in public healthcare, make upgrades difficult, creating a gap with the private sector. This was observed in both the literature and the focus group. Failing to adequately maintain and upgrade IT infrastructure components, including hardware and software, creates critical vulnerabilities that cybercriminals can exploit to gain unauthorised access to sensitive healthcare data [34]. Even a bad experience, the cost versus benefits, and the installation process are all factors in a user’s reluctance to update software, operating systems and hardware [47]. This was supported by the literature.

This lack of investment can have devastating consequences. Outdated software might contain known security flaws that hackers can easily manipulate. Similarly, ageing hardware can malfunction, leading to data breaches or system outages. These breaches can expose medical records and financial details. In the worst-case scenario, a cyberattack could compromise patient data, delay diagnoses, hinder treatment plans, and ultimately endanger patient safety. Coventry and Branley [27] found that the use of legacy systems contributed to the high incidence of cybersecurity attacks in the healthcare sector.

While some participants in the focus group downplayed the inherent risk of legacy systems, others highlighted the increasing sophistication of cybercriminals who can exploit any vulnerability. This necessitates continuous investment in security measures to stay ahead of evolving threats. The perception of IT security as an “indirect overhead” rather than a core clinical service further marginalises its importance in budget allocation decisions. This mindset overlooks the potential financial consequences of a cyberattack, which can be far greater than preventative measures.

Investment in cybersecurity technology, personnel, training, incident response and regulatory compliance can vary greatly depending on the healthcare organisation’s size and funding model. The amount allocated to this area is also often in competition with the service delivery of healthcare or only a small amount of their total ICT budget [48]. As discussed in the focus group, effective cybersecurity requires a comprehensive approach that includes user training alongside technological solutions. However, the ongoing cost of training and maintaining security protocols can be discouraging, leading to a defeatist attitude among some users. Unfortunately, cyberbreaches only exacerbate the healthcare industry’s financial struggles due to high expenses and low-profit margins compared to other sectors [17].

Integrating medical devices with networks (IoT) and allowing personal devices (BYOD) creates new attack points that are not always considered. This was supported by both the literature and the focus group. The open environment of some healthcare organisations also makes it difficult to control device usage and secure entry points, creating a conflict with robust cybersecurity practices. Simply the vast number of users accessing data, from patients to professionals, complicates access control.

Legacy legislation and internal healthcare organisation policies and practices struggle to keep pace with evolving technology. Some current practices prioritise convenience, like non-expiring passwords and guest Wi-Fi, creating vulnerabilities under the guise of user-friendliness. Whilst the need to update existing legislation and regulations to reflect the evolving nature of cyber threats was thought to be important, concerns were raised about the adequacy of existing standards and enforcement mechanisms.

While acknowledging the importance of robust cybersecurity, participants emphasised the financial strain it places on healthcare organisations. This makes it difficult to afford necessary upgrades, software updates, and skilled IT security personnel who often exceed cybersecurity budgets. Given the evolving cyber threat landscape, participants also argued that healthcare (especially public healthcare) cannot address these challenges alone and that there is a need for increased government funding.

Australian legislation emphasises the importance of patient privacy and data confidentiality. However, participants acknowledge the high value of patient data on the black market and a general lack of public understanding regarding how healthcare data is stored and accessed. A culture of secrecy surrounds cyberattacks in healthcare. While some participants believe transparency is crucial for public awareness and improvement, others worry it could erode trust in the healthcare system.

Often, the public is made aware of cybersecurity incidents through major media reports, blog articles and social media posts [49]. Research conducted in other fields, such as the stock market, suggests that when an incident occurs, there is a temporary adverse reaction (e.g. a temporary drop in stock prices) once the incident is reported through public media; however, this does not last long [50]. Wang et al. [50] also surmised that being vague or perceived as ‘hiding’ a security breach imposed a higher level of negative perception from the public.

The media’s role in shaping public perception is significant. Open communication about cyberattacks can force improvements, but sensationalised media coverage can lead patients to fear the security of their data. Media exposure to cybersecurity incidents can be significant for education and protection strategies. It also can distort perceived cybersecurity risk by sensationalising low-likelihood attacks [51]. Transparent communication about cyberattacks in the media was viewed as beneficial as it can raise public awareness, encourage user education, and pressure policymakers to address the issue.

Participants stressed the need to move away from reactive measures and take a more proactive stance against cyberattacks. Educating those within the healthcare sector regarding cyber threats and best practices was seen as crucial, alongside clear communication and education about the benefits of initiatives like My Health Record, to ensure patients feel empowered and secure when engaging with their health data. Since human behaviour is often seen as the most vulnerable point in an organisation’s cybersecurity defences [34], implementing robust countermeasures specifically targeting user behaviour is crucial. This necessitates prioritising education and training programs. Participants also stressed the importance of finding a cost-effective balance to ensure trust and data security, with an emphasis on the need for a robust and publicly accessible framework specifically tailored to the Australian healthcare sector.

## Future direction

The findings from the literature review and the focus group discussions converged on a critical need for improved healthcare cybersecurity. While the literature provided a comprehensive understanding of existing threats and vulnerabilities, the focus group discussions offered a more practical Australian perspective. Both sources highlighted the challenges posed by inadequate investment in cybersecurity measures. Furthermore, both the literature and the focus group participants emphasised the importance of user training and awareness programs to address the human element of cyberrisk. However, a key takeaway message emerged: the need for a proactive rather than reactive approach. Considering the financial constraints and the human element in cybersecurity, the concept of a self-assessment tool surfaces as a potential solution.

Sardi et al. [38] underscore the critical need for robust risk assessment methodologies in their comprehensive review of healthcare cybersecurity literature. While such research plays a vital role in illuminating existing threats and vulnerabilities, it is crucial to translate these findings into actionable strategies for the healthcare sector. Herein lies the potential value of an Australian healthcare cybersecurity self-assessment tool.

By pinpointing vulnerabilities and cybersecurity gaps, such a tool could empower the healthcare sector to priortise resources and build stronger defences. This targeted approach could significantly improve the sector’s preparedness.

Undertaking a cybersecurity self-assessment (also known as a cybersecurity index) can be useful for an organisation to determine how prepared it is against potential security threats. An index typically includes a range of questions organised into categories and designed to cover all the critical aspects of an organisation. By completing these assessments, users better understand where their organisation stands in terms of its ability to fend off cyber-threats and protect sensitive data.

**Fig. 1:**
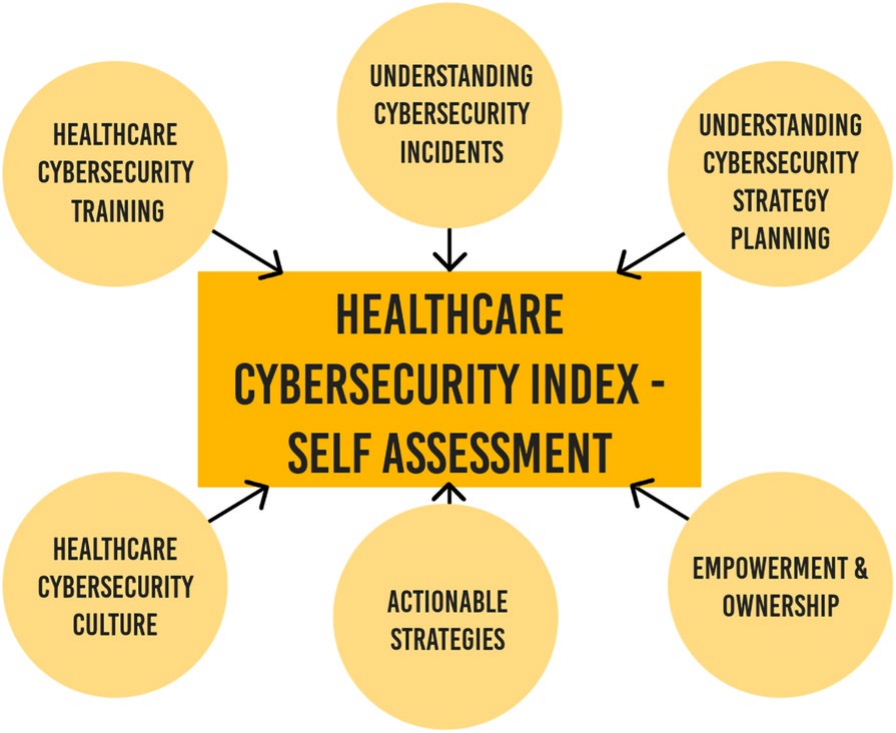
Components needs in a Healthcare Cybersecurity Index - Self Assessment

However, self-assessments are often generic. Most commonly, they cover cyber-response readiness, resilience readiness, governance and leadership at an organisational or government level [52]. While it might be feasible for a generic pre-existing cybersecurity index to work with the healthcare sector, further research is needed. Any self-assessment tool will need to address the points raised in this article. Figure 1 shows a summary of the key components that would need to be considered when designing a self-assessment for the Australian healthcare sector.

The development of an Australian healthcare cybersecurity self-assessment tool would also empower the sector to take ownership of its cybersecurity position while also providing a standardised approach to cybersecurity assessment. This, in turn, would represent a significant step towards a more cyber-secure healthcare environment in Australia. Hassandoust and Techatassanasoontorn [53] found that individuals who possess a sense of empowerment are highly driven to carry out a diverse range of proactive actions connected to their responsibilities.

Ultimately, being proactive and empowered in cybersecurity is crucial for staying one step ahead of cyber-threats, protecting sensitive data, maintaining business operations, complying with regulations, and building trust with stakeholders. In addition, it allows organisations to identify vulnerabilities early on, implement appropriate security measures, and respond effectively to potential cyber-incidents.

## Limitations

This study was focused on one Australian state, Victoria, with most participants familiar with regionally based Victorian healthcare services. Therefore, caution should be exercised when applying these themes to Australia’s metropolitan or remote healthcare services.

As with any focus group, there is a chance that participants gave replies they anticipated would be favoured by their peers. However, given the sensitive nature of cybersecurity and healthcare, participants were likely to have responded conservatively due to legal and perception factors.

Finally, while a sample size of five participants did provide valuable insights, a larger sample population could offer a more comprehensive understanding of the current cybersecurity issues faced by Australian healthcare services. Additionally, with a broader participation pool, we could explore a wider range of perspectives that this study may not have fully captured.

## Conclusion

The Australian healthcare sector faces a growing threat from cyberattacks, consistently reporting the highest number of data breaches compared to other industries [1–3]. The shift to remote work due to COVID-19 has further exposed vulnerabilities in healthcare cybersecurity [8].

The consequences of cyberattacks in healthcare can be devastating. Compromised patient data can have a severe impact on individuals, while disrupted operations can hinder healthcare provision. Furthermore, the financial toll of a cyberattack can be enormous, with expenses from data recovery to reputational damage significantly impacting healthcare budgets.

This paper explores these challenges, highlighting the financial strain of maintaining outdated systems, outdated legislation, the evolving nature of cyber threats, and the complexities of the healthcare environment itself. Even balancing patient privacy and public awareness of cyberattacks is a delicate dance. While transparency is crucial for raising public awareness and encouraging user education, it can also erode trust in the healthcare system if not handled carefully. Media coverage of cyberattacks can play a role in raising awareness, but sensationalised reports can lead to panic and distrust.

The paper highlights the need for a proactive approach to cybersecurity in the Australian healthcare sector, emphasising the need for cybersecurity education for those in the sector and encouraging empowerment. The development of a tailored self-assessment tool would help the sector identify vulnerabilities and effectively prioritise its cybersecurity resources. A self-assessment would also help understand what “business as usual” looks like. For example, does the healthcare organisation have a cybersecurity strategy, are employees aware of cybersecurity risks, and what training is available to help prevent and mitigate risk? It can also potentially give the user strategies to help build a better defence against cyberattacks and supports self-efficacy, the confidence or strength of belief that an individual has to execute behaviours necessary to reach attainment [54].

This research is an initial step towards a more comprehensive project: developing an Australian Cybersecurity Healthcare Index - Self Assessment. The valuable insights from this study will form a foundational block for this future index. By incorporating the findings here with data from additional research, we aim to create a self-assessment tool that will empower Australian healthcare institutions to evaluate their cybersecurity preparedness. This index will ultimately contribute to a more secure healthcare landscape for Australians.

## Data Availability

The raw data is not publicly available to preserve individuals’ privacy and to adhere to the stipulations outlined in the ethics approval (B19-087). Upon reasonable request, the de-identified data is available from the corresponding author (W.B.).
